# Hemiarthroplasty as a Viable Option for Unstable Intertrochanteric Femur Fractures in the Elderly: A Functional Outcome Analysis

**DOI:** 10.7759/cureus.100458

**Published:** 2025-12-30

**Authors:** Ishmita Paul, Akshay Ks, Amit K Yadav, Sangeet Gawhale

**Affiliations:** 1 Trauma and Orthopaedics, Hillingdon Hospital, London, GBR; 2 Orthopaedics and Traumatology, Peninsula Orthopaedic Research Institute, Sydney, AUS; 3 Orthopaedics and Traumatology, Ulster Hospital, Belfast, GBR; 4 Orthopaedics and Trauma, Grant Government Medical College and Sir JJ Group of Hospitals, Mumbai, IND

**Keywords:** bipolar hemiarthroplasty, extracapsular neck fracture, hemiarthroplasty of the hip, neck of femur fracture, unstable intertrochanteric femur fracture

## Abstract

Background: Unstable intertrochanteric femur fractures in elderly osteoporotic patients are associated with high morbidity and mortality. While internal fixation remains the standard treatment, fixation failure and prolonged immobilisation are frequent concerns. Primary cemented bipolar hemiarthroplasty offers the potential for immediate stability and early mobilisation, reducing postoperative complications.

Methods: This prospective study included 30 elderly patients (>60 years) with unstable intertrochanteric fractures treated with primary cemented bipolar hemiarthroplasty from June 2021 to August 2024. Functional and radiological outcomes were assessed using the Harris Hip Score and radiograph at regular intervals.

Results: The mean operative time was 81 minutes with an average blood loss of 361.03 ml. Full weight-bearing was achieved at a mean of 3.2 days postoperatively. At one year, 76% of patients demonstrated fair to excellent outcomes on the Harris Hip Score. Limb length discrepancy (<2 cm) was noted in 10 cases, primarily due to prosthetic subsidence from calcar loss. The overall mortality rate was 16.6%, with all deaths occurring due to causes unrelated to surgery. No cases of dislocation were observed.

Conclusion: Primary cemented bipolar hemiarthroplasty is a safe and effective option for managing unstable intertrochanteric femur fractures in elderly osteoporotic patients. It allows early mobilisation, restores function, and minimises complications related to prolonged recumbency. Larger comparative studies with longer follow-up are needed to further validate its long-term efficacy over internal fixation.

## Introduction

Intertrochanteric femur fractures are common in the elderly, typically resulting from low-energy trauma [1]. Their incidence continues to rise globally due to increased life expectancy and osteoporosis, accounting for nearly 50% of all hip fractures. Although the one-year mortality rate has declined compared to earlier reports, it remains high at 20-27% [2,3].

While stable intertrochanteric fractures are effectively managed with internal fixation, the optimal treatment of unstable patterns in osteoporotic bone remains controversial [4]. Conventional fixation techniques, including dynamic hip screws (DHS) and intramedullary nails, have shown limited success in managing unstable intertrochanteric fractures in osteoporotic patients. The compromised trabecular and cortical bone quality in these individuals reduces implant anchorage and mechanical stability, predisposing them to complications such as screw cut-out, implant migration, and excessive fracture collapse [5,6]. Moreover, restricted postoperative mobilisation increases the risk of complications such as pneumonia, pressure sores, and deep vein thrombosis in this frail population [7,8].

Primary hemiarthroplasty has emerged as a viable alternative for managing unstable intertrochanteric femur fractures in elderly patients, offering the advantages of early full weight-bearing and faster rehabilitation. However, concerns persist regarding cement-related complications and outcomes. This study aims to evaluate the functional outcomes of primary hemiarthroplasty in this patient population.

## Materials and methods

This prospective study was conducted in the Department of Orthopaedics of Grant Government Medical College and Sir JJ Group of Hospitals, Mumbai, India, from June 2021 to August 2024 after obtaining approval from the same institution (approval number: IEC/PG/479/Dec/2018). Thirty elderly patients (>60 years) with unstable intertrochanteric femur fractures treated with primary hemiarthroplasty were included. 

Inclusion and exclusion criteria

Patients aged above 60 years with unstable intertrochanteric fractures (Evans type III or higher), reverse oblique patterns, or failed internal fixation were included. In contrast, excluded were patients below 60 years, unfit for anaesthesia, and with stable intertrochanteric fractures (Evans I-II), pathological fractures, long preoperative recumbency, refusal for procedure, pre-existing hip pathology, and previous hip surgeries.

All patients underwent detailed clinical evaluation, including history, physical examination, and radiographic assessment (anteroposterior pelvis with both hips and cross-table lateral views). Routine laboratory investigations and medical optimisation were performed. 

Outcome assessment

Functional Evaluation

Functional outcomes were assessed using the Harris Hip Score (HHS), a validated outcome measure, recorded preoperatively and at each follow-up interval. The HHS, an open-access validated outcome measure, was used to assess functional outcome [9].

Gait Assessment

Postoperative gait was assessed at each follow-up appointment by both the treating orthopaedic surgeon and the physiotherapist. Patients' mobility status was clinically classified into one of the following categories: walking independently without any aid, walking with the assistance of a stick, walking with the support of a walker, being unable to mobilise and confined to bed, or having passed away during the follow-up period.

Radiological Evaluation

Serial radiographs assessed implant position, subsidence, heterotopic ossification, and acetabular erosion.

Statistical analysis

Statistical analysis was performed using IBM SPSS Statistics for Windows, Version 25.0 (IBM Corp., Armonk, New York, United States). Continuous variables were expressed as mean±standard deviation and categorical data as frequency and percentage. Preoperative and postoperative HHS were compared using the paired t-test, which demonstrated a statistically significant improvement in functional outcome (p<0.001). Gait status at the final follow-up was compared using the chi-squared test, which showed a considerable improvement in ambulatory ability following surgery (p<0.05).

Aim and objectives

This study aimed to evaluate the functional outcomes of primary hemiarthroplasty for unstable intertrochanteric femur fractures in elderly patients. The objectives were to assess postoperative pain relief, the ability to perform activities of daily living, time to early ambulation, incidence of postoperative complications, and the need for revision surgery.

Surgical technique

All patients were medically optimised preoperatively. Standard aseptic preparation and antibiotic prophylaxis were performed. Surgery was conducted under spinal or general anaesthesia using the posterior approach with the patient in the lateral decubitus position.

**Figure 1:**
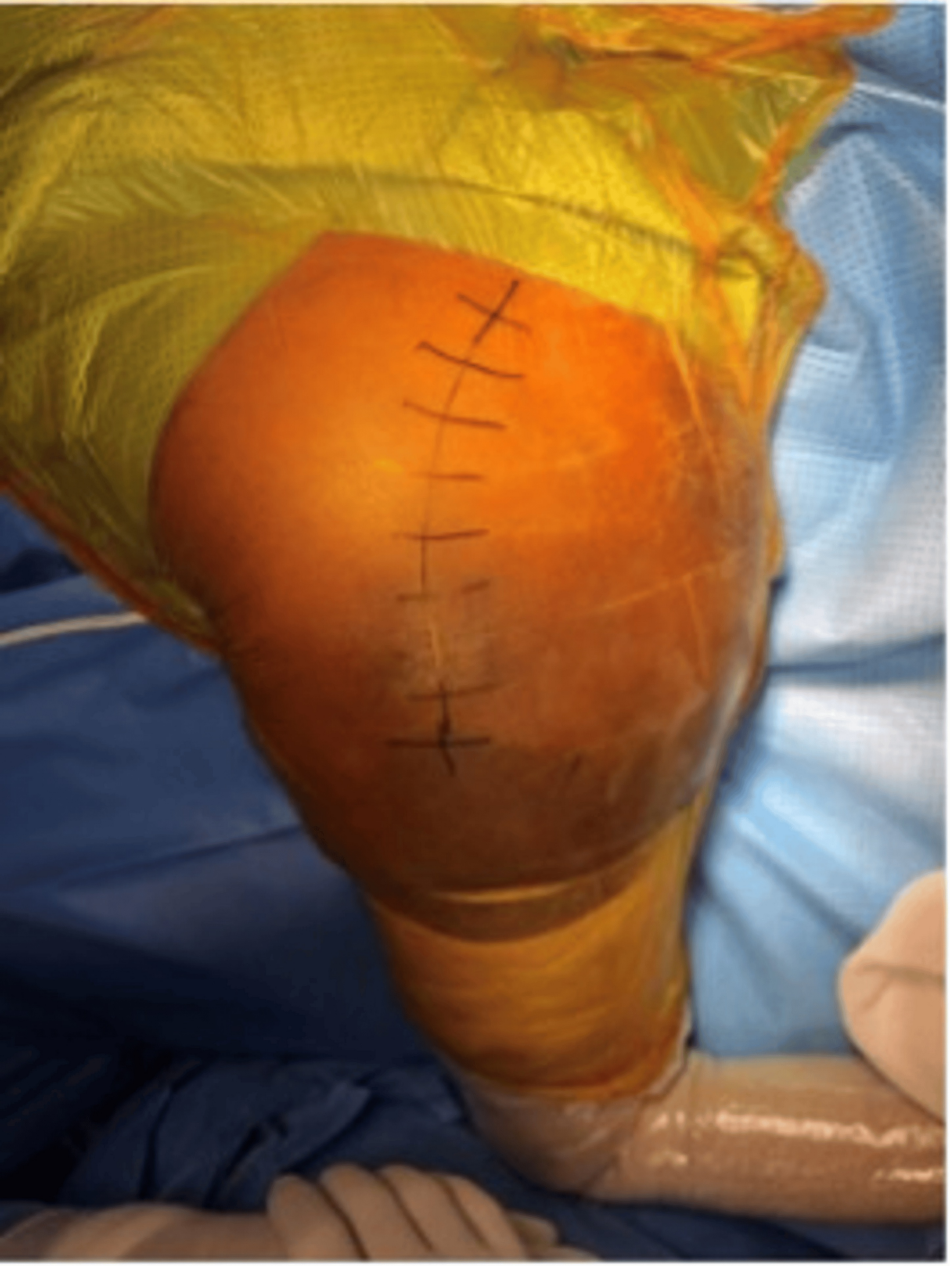
A photograph demonstrating the posterior approach to the hip in the lateral decubitus position, showing the skin incision centred over the posterior aspect of the greater trochanter

**Figure 2:**
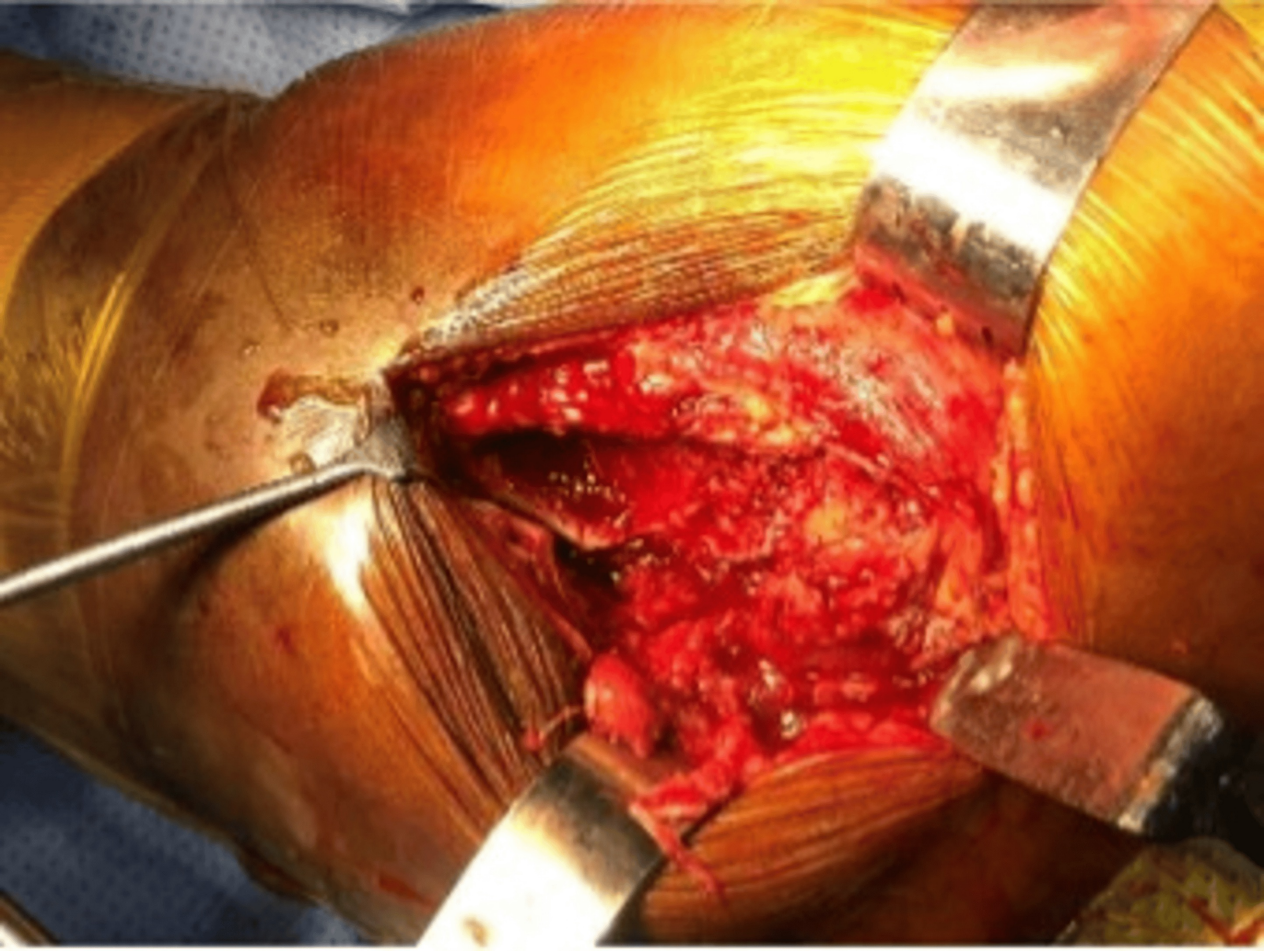
Approach through the fracture line

The incision was made starting 10 cm distal to the posterior superior iliac spine, extending distally and laterally along the posterior margin of the greater trochanter, and then directed approximately 10 cm parallel to the femoral shaft (Figure 1). The deep fascia was exposed, and the gluteus maximus muscle was split along the line of its fibres using blunt dissection. By retracting the proximal fibres superiorly and the distal fibres inferiorly, the greater trochanter was exposed. The fractured greater trochanter was reflected anteriorly to improve access, and the deeper dissection was carried out through the fracture site (Figure 2). The sciatic nerve was not routinely exposed. After exposing the posterior capsule, a T-shaped capsulotomy was performed. The thigh and knee were flexed to 90°, and the hip was internally rotated to expose the femoral neck. Osteotomy was then performed at the level of the femoral neck, and the femoral head was extracted from the acetabulum. The head's size was measured using a template. The acetabulum was prepared by excising the remnant ligamentum teres and curetting the remaining soft tissue from the pulvinar region.

**Figure 3:**
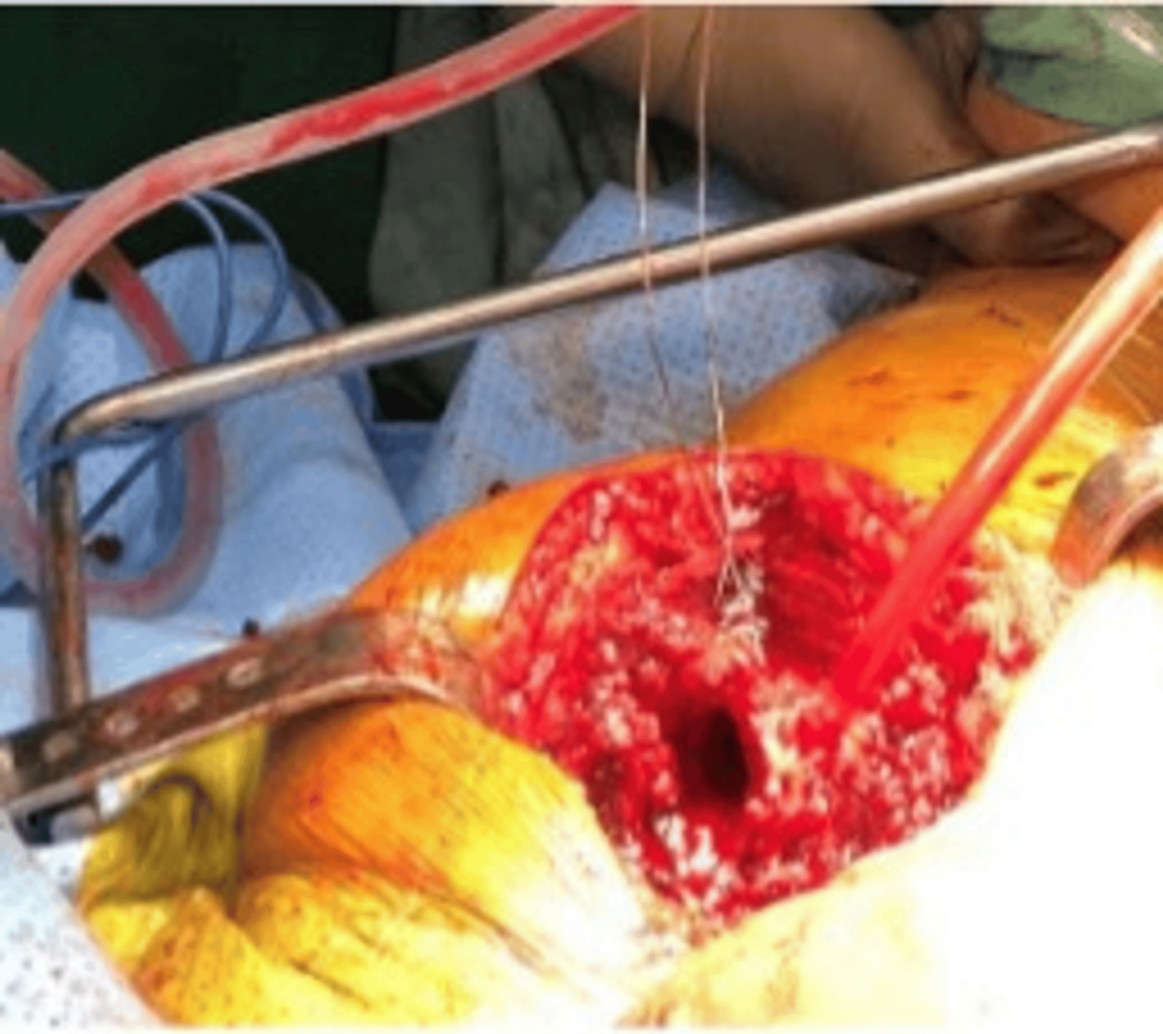
Distal limb of tension band wiring secured to the shaft

**Figure 4:**
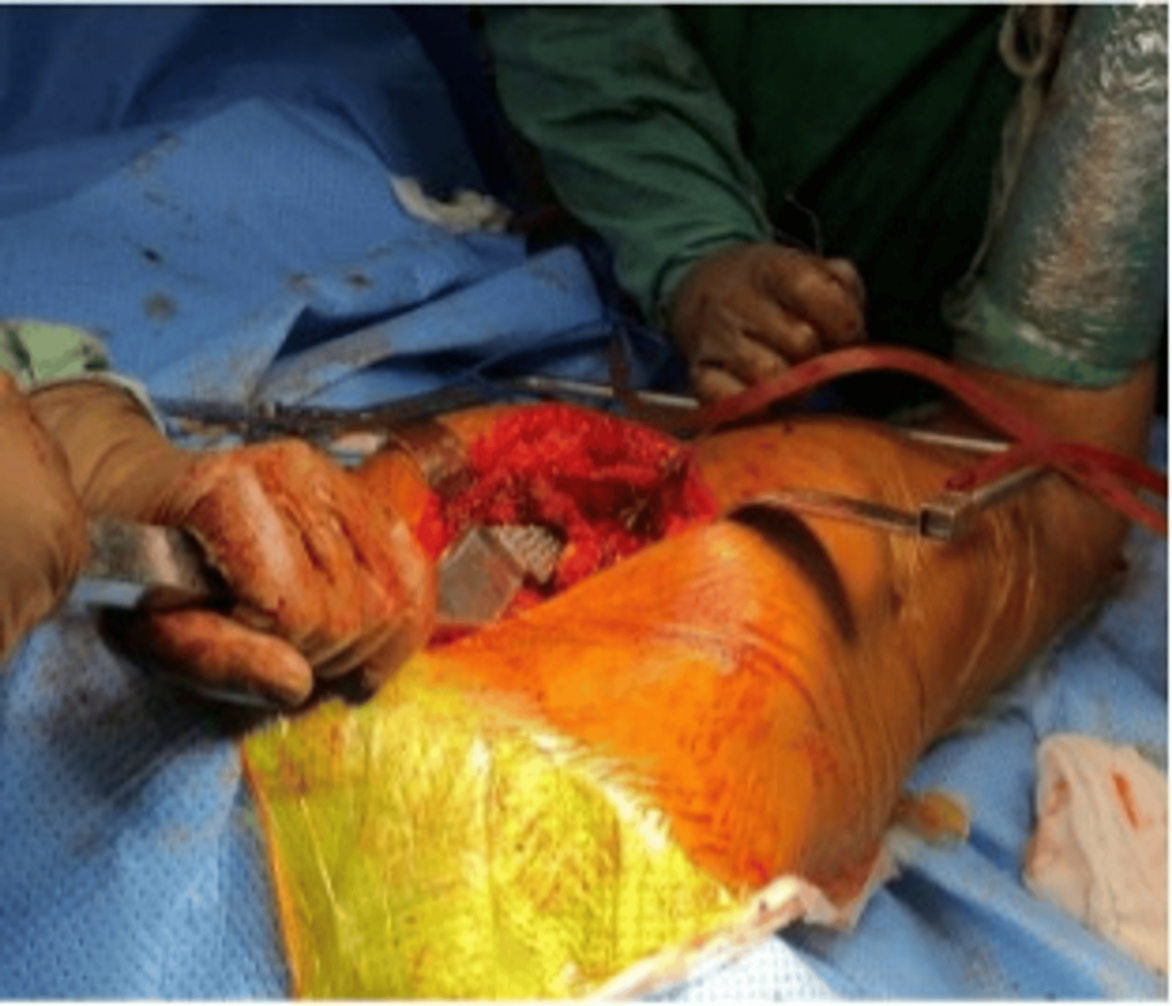
Femoral canal preparation with 15° anteversion

The distal limb of tension band wiring is secured to the shaft to prevent fracture propagation (Figure 3). The preparation of the femoral canal began with a liberaliser to prevent varus alignment of the stem. Sequential broaching was performed until an appropriate fit was achieved for prosthesis insertion (Figure 4). All patients had Dorr type C femurs; therefore, a standard cementing technique with a cement restrictor was used in all cases. With the knee and tibia held vertically, femoral stem anteversion was adjusted to 10-15°. The prosthesis was inserted into the femoral canal at 90° to the axis of the vertical tibia and 10-15° anteriorly to achieve the desired anteversion.

**Figure 5:**
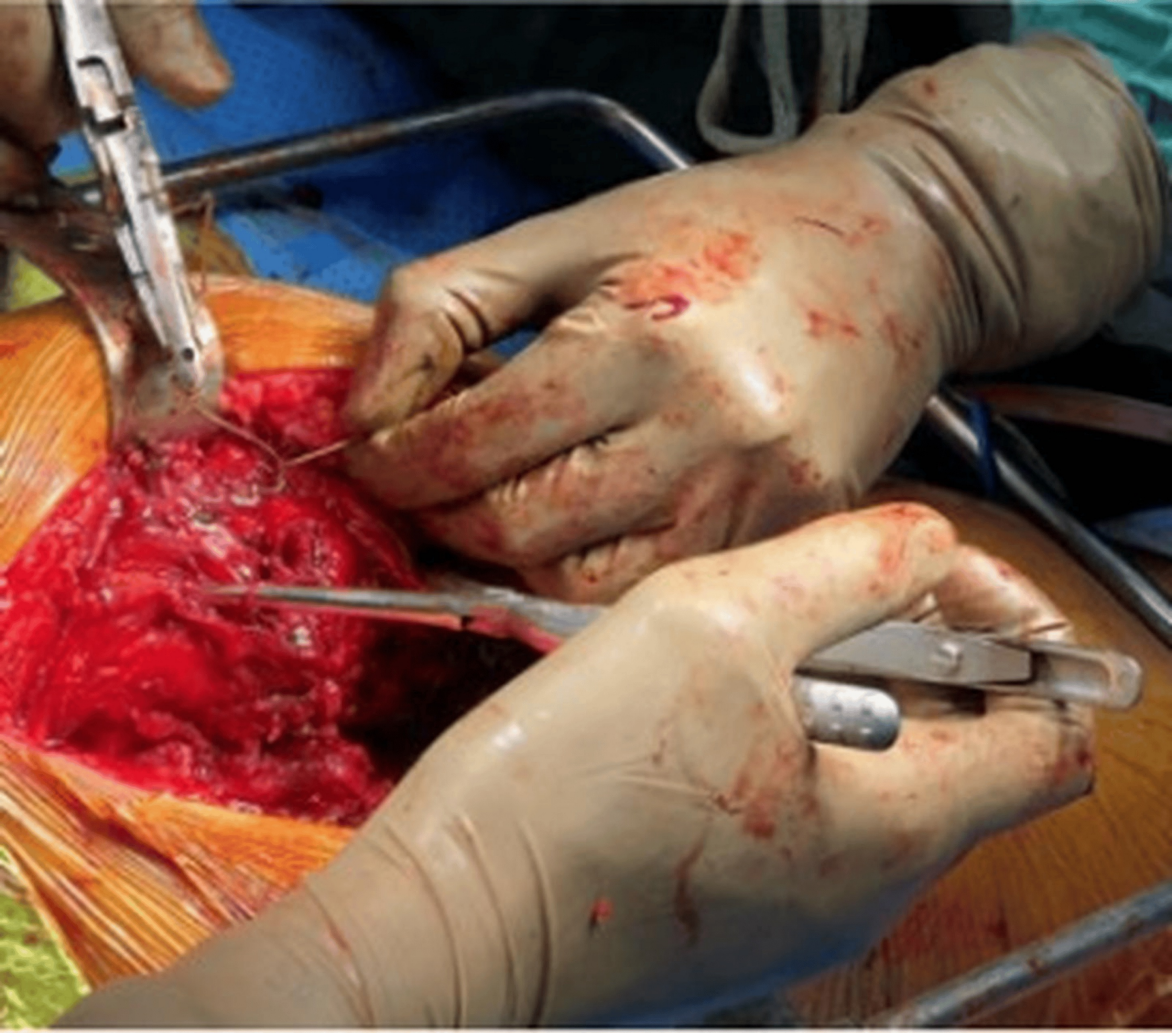
Femoral head reduction into the acetabulum

**Figure 6:**
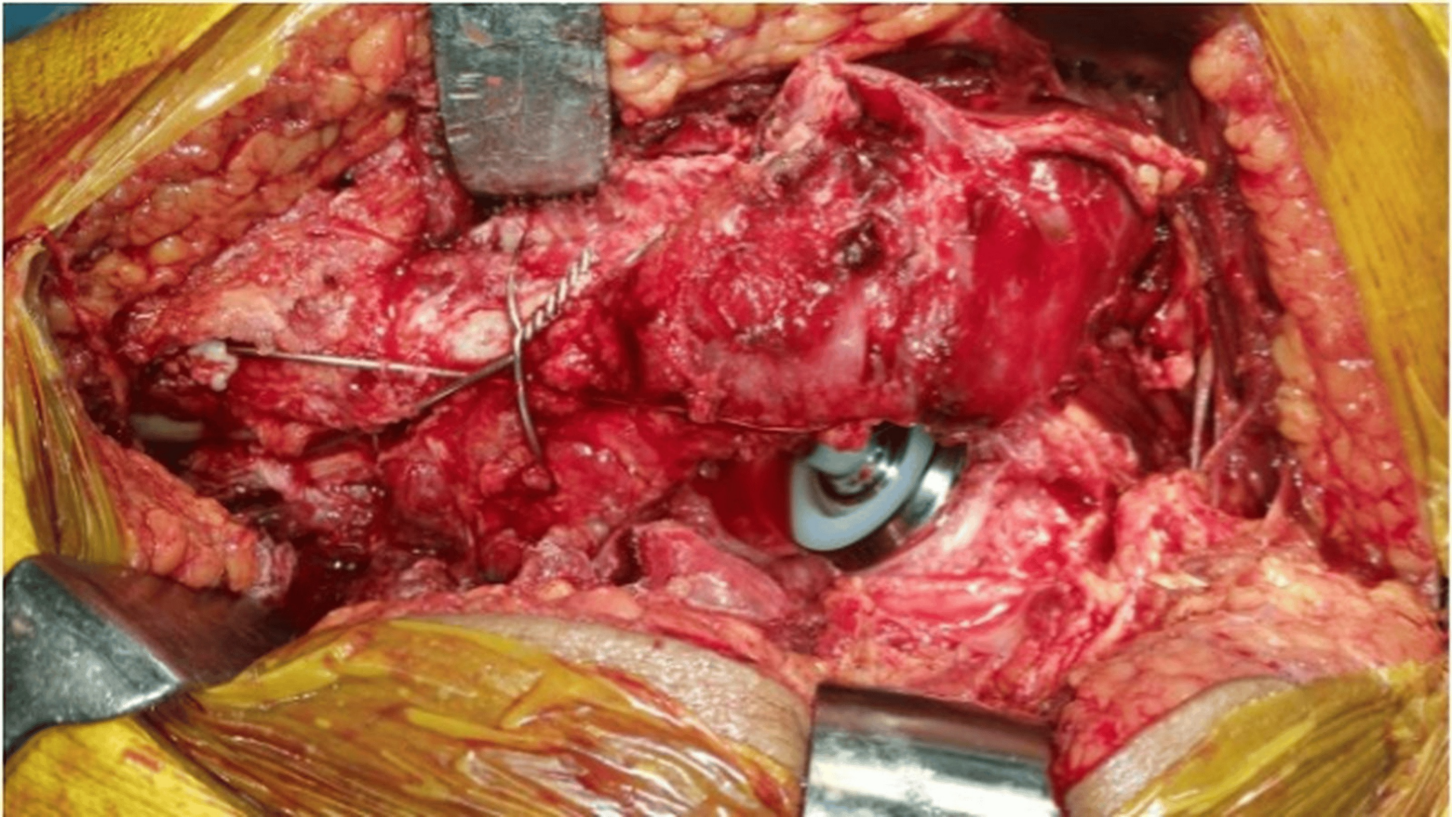
Anatomical reduction and final tightening of tension band wiring around the greater trochanter

The greater trochanter was repositioned and fixed with cerclage wires. If the lesser trochanter was fractured, it was similarly reduced and secured using cerclage wiring (Figure 5). In selected cases, additional fixation was performed using a Kirschner wire combined with tension band wiring or a reconstruction plate, depending on fracture configuration, bone quality, and intraoperative stability. In cases with minimal displacement and satisfactory stability, no trochanteric fixation was undertaken. A trial reduction was then performed to assess leg length equality and ensure appropriate abductor muscle tension. After confirming the correct femoral head size, the final implant was positioned and reduced into the acetabulum (Figure 6). The capsule was closed, followed by the repair of the external rotators. The wound was then closed in layers.

Postoperative protocol

Patients were mobilised and allowed full weight-bearing ambulation with a walker on the same day postoperatively, depending on pain tolerance. All patients had a minimum follow-up of six months. Surviving patients were followed up to 12 months, and final functional and radiological outcomes were analysed at the one-year follow-up where available. Functional outcomes were assessed using the HHS, while radiological evaluation included the assessment of stem alignment, loosening, and periprosthetic fractures based on standard zonal analysis.

## Results

**Figure 7:**
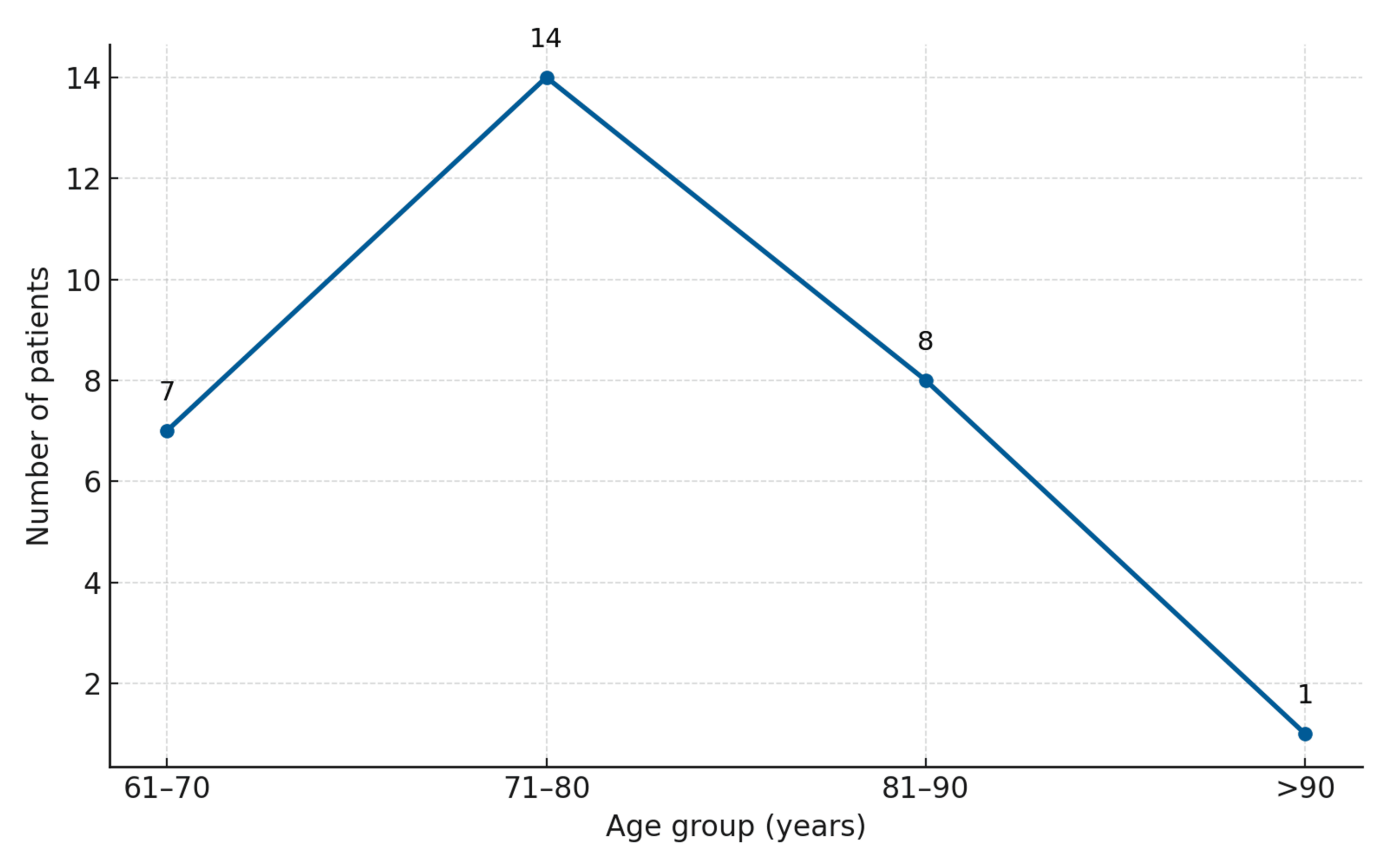
Age distribution of patients with intertrochanteric fractures treated with bipolar hemiarthroplasty The y-axis represents the number of patients, and the x-axis represents age groups in years. The line graph illustrates the distribution of patient ages across four groups: 61-70 years, 71-80 years, 81-90 years, and above 90 years. The majority of patients (n=14) were in the 71-80-year age range, followed by eight patients aged 81-90 years, seven patients aged 61-70 years, and one patient over 90 years of age.

**Figure 8:**
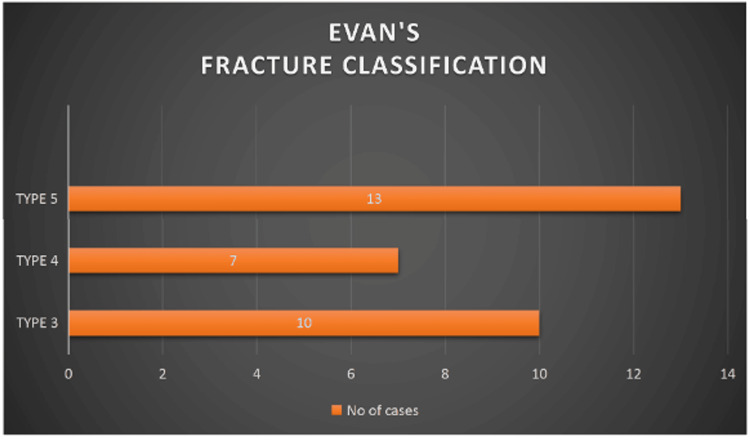
Distribution of intertrochanteric fractures based on Evan's classification

A total of 30 patients with intertrochanteric fractures were treated with cemented bipolar hemiarthroplasty. The mean age of the cohort was 76.43 years (range 65-90 years). The study included 15 males and 15 females, with a 1:1 male-to-female ratio (Figure 7). The majority of fractures (16 cases) were on the right side, while 14 involved the left side. According to Evan's classification, 10 patients (33.3%) had type III fractures, seven patients (23.3%) had type IV fractures, and 13 patients (43.3%) had type V fractures (Figure 8).

The mean duration from injury to surgery was six days. All procedures were performed using a posterior approach. The mean operative time was 81 minutes, with an average intraoperative blood loss of 361.03 ml. 

Preoperatively, five patients (16.7%) required blood transfusions, and intraoperatively, none of the patients required transfusions; all procedures were uneventful. The mean postoperative hospital stay was 11.3 days. Full weight-bearing was initiated at a mean of 3.2 days postoperatively (range 2-10 days).

**Figure 9:**
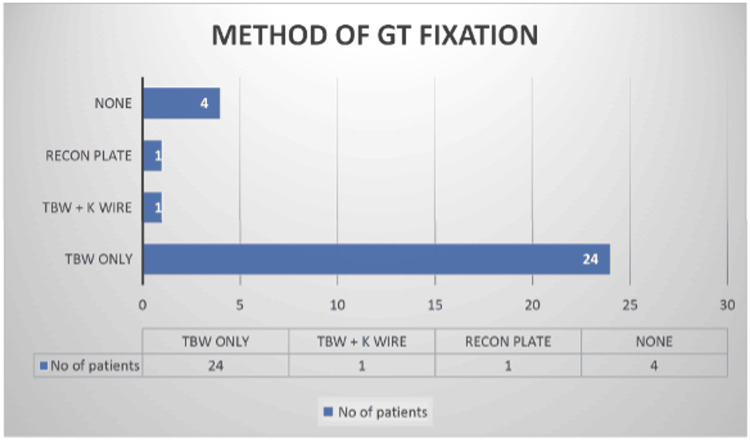
Methods used for fixation of the GT used in the study The majority of patients underwent fixation with TBW alone (n=24), followed by TBW with a K-wire (n=1), a reconstruction plate (n=1), and no fixation (n=4). GT: greater trochanter; TBW: tension band wiring; K-wire: Kirschner wire

Various techniques were used to fixate the greater trochanter. Tension band wiring alone was performed in 16 patients, Kirschner wire with tension band wiring in one case, and reconstruction plating in another. In four patients, no fixation was attempted (Figure 9).

**Figure 10:**
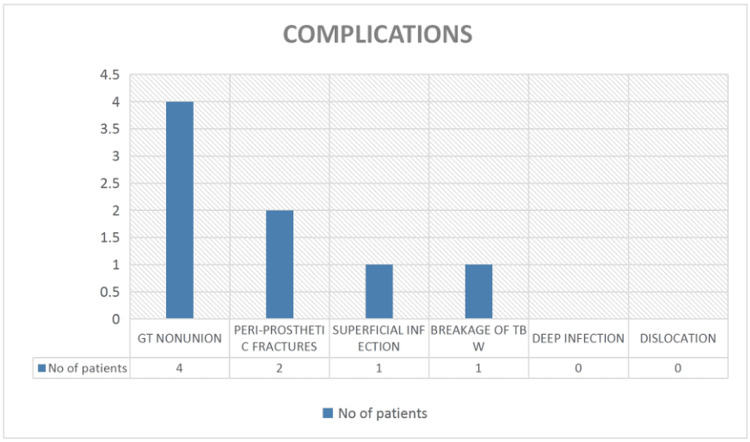
Distribution of postoperative complications following bipolar hemiarthroplasty for intertrochanteric fractures

Comorbidities and postoperative complications were noted among the study population. Hypertension was the most common comorbidity, followed by type 2 diabetes mellitus. Ten patients had a single comorbid condition, while eight had multiple comorbidities, including bronchial asthma, chronic renal failure, tuberculosis, and epilepsy. Greater trochanter nonunion occurred in four patients, resulting in residual pain and abductor weakness; three of these patients were bedridden and required assistance for ambulation, while one was ambulatory with a walker. One patient developed a superficial infection that resolved with intravenous antibiotics. Two patients sustained periprosthetic fractures: one managed with plate fixation and the other requiring stem removal and replacement with a long K-nail and cerclage wiring. The latter subsequently underwent implant removal due to K-nail dislocation. Additionally, one patient experienced cerclage wire breakage, leading to persistent pain and limited mobility (Figure 10).

Five patients (16.7%) died within six months postoperatively due to causes unrelated to surgery. The remaining 25 patients were followed up at three, six, and 12 months. Among these, 10 patients demonstrated a limb length discrepancy of less than 2 cm, which was effectively managed with the use of a heel raise. One patient, however, had a shortening exceeding 2 cm, leading to a noticeable limp and progressive functional deterioration, ultimately resulting in a bedridden state.

**Figure 11:**
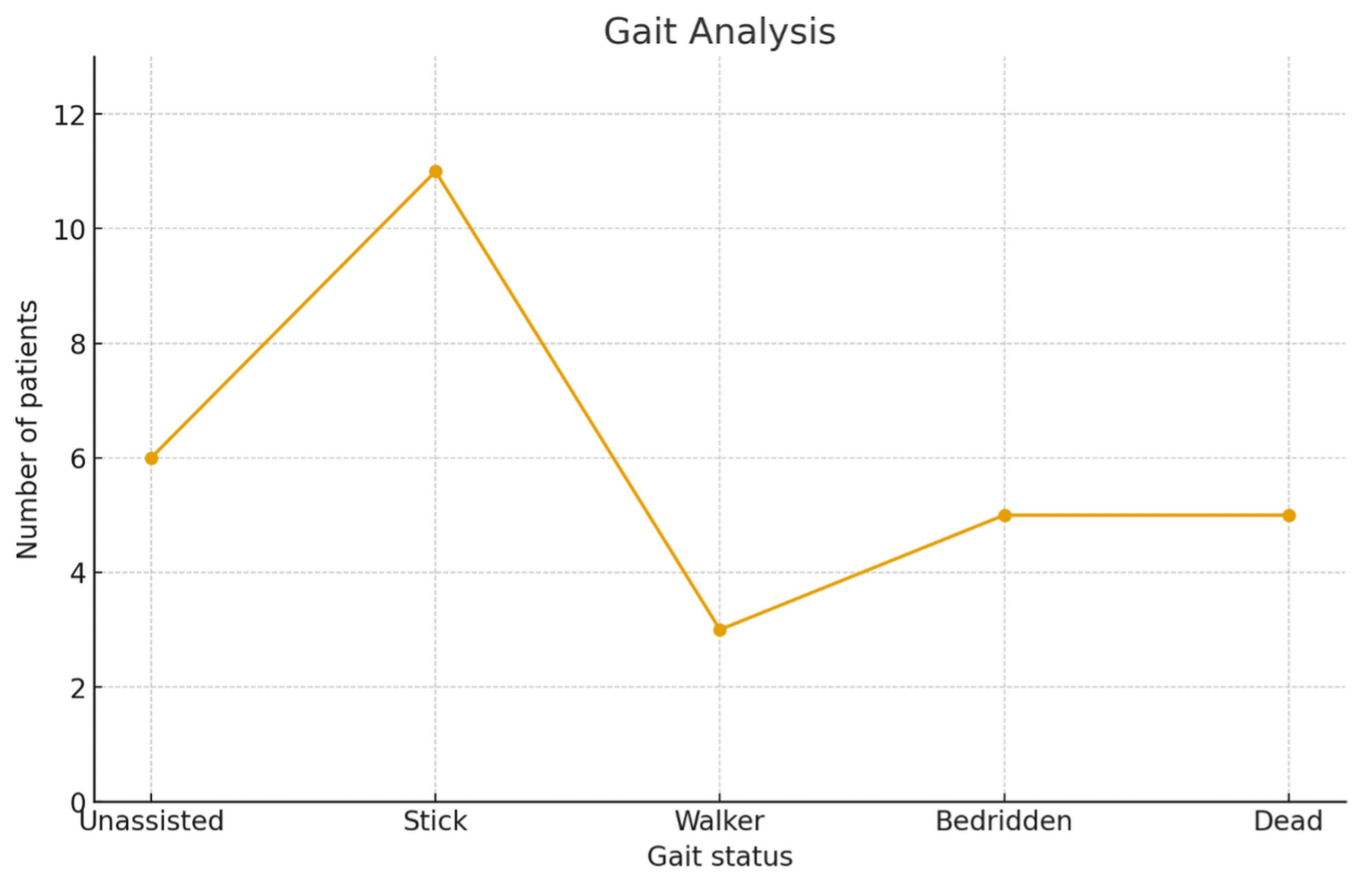
Gait analysis at the 12-month follow-up

At the end of 12 months, four patients were able to walk without any support, while 11 patients ambulated with the aid of a cane or stick, and three patients required a walker for mobility (Figure 11). Eleven patients were able to ambulate independently. In our study, five patients were bedridden, and another five died due to causes unrelated to surgery, accounting for an overall mortality rate of 16.6%.

**Figure 12:**
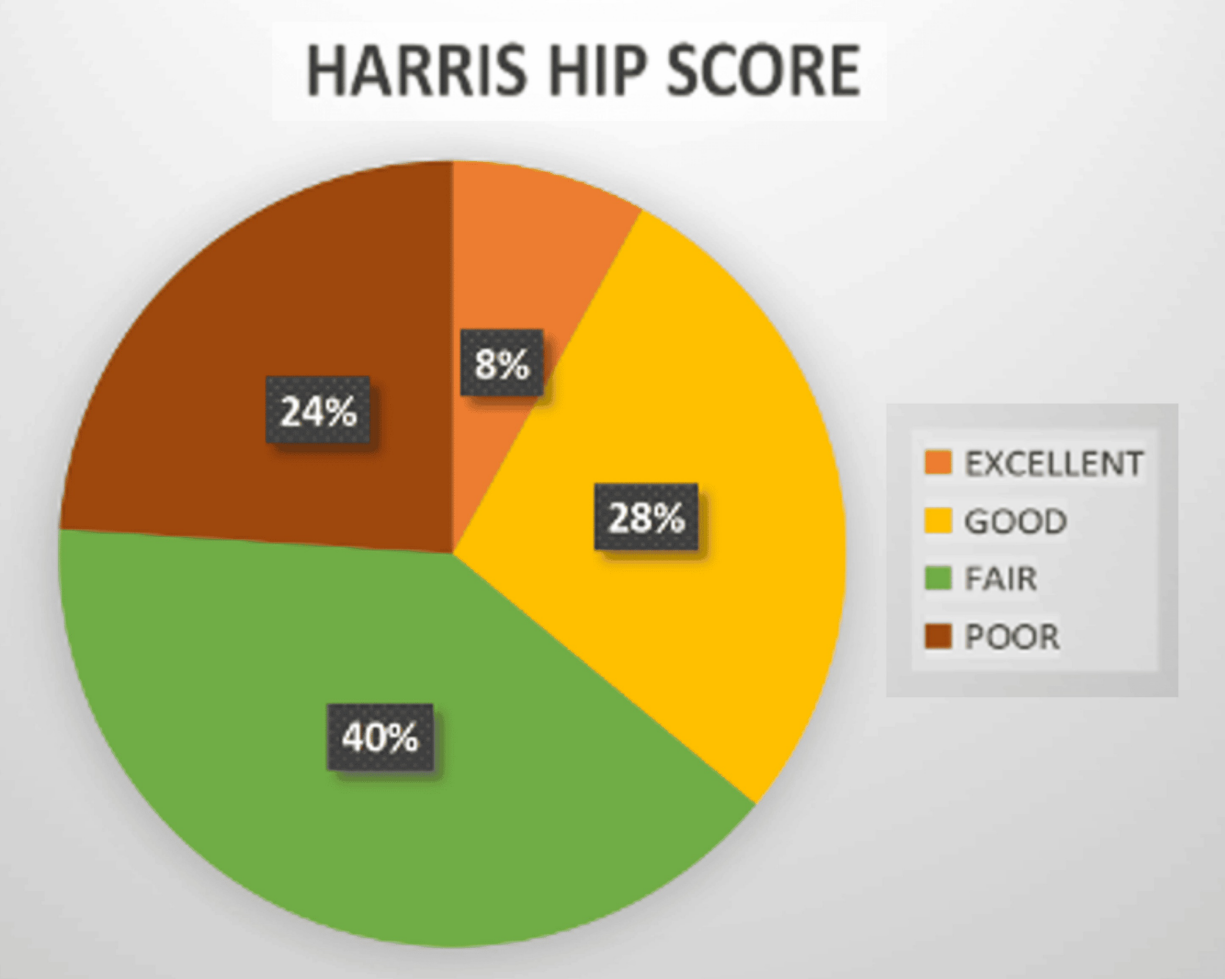
Distribution of functional outcomes based on the Harris Hip Score

The functional outcomes were assessed using the HHS. In this study, two patients achieved excellent results, seven had good results, 10 had fair results, and six demonstrated poor outcomes. Among the 25 patients who were alive and available for follow-up assessment, 19 (76%) had excellent to fair results on the modified HHS (Figure 12).

**Figure 13:**
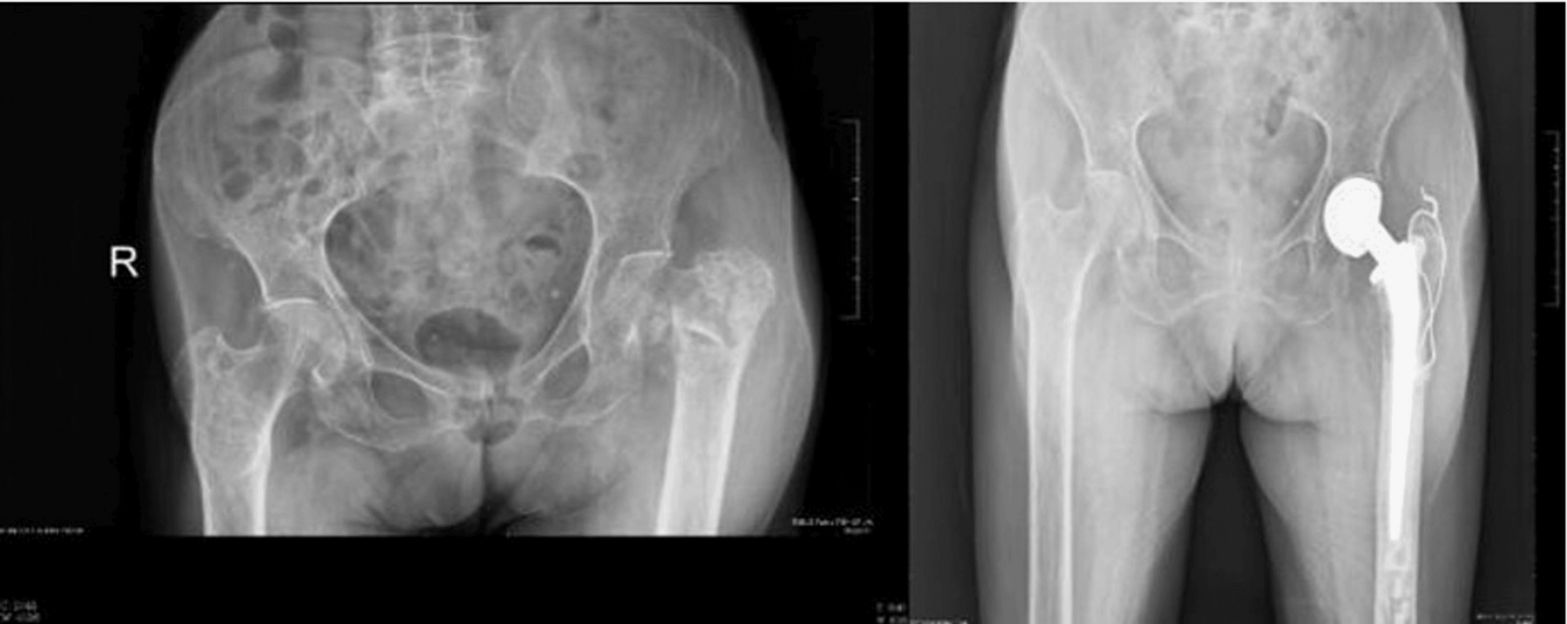
Preoperative and postoperative radiographs Preoperative anteroposterior radiograph of the pelvis showing a right-sided intertrochanteric femur fracture (left image). Postoperative radiograph at the one-year follow-up demonstrating satisfactory alignment, adequate stem positioning, and stable fixation (right image)

In this study, the bipolar stem was positioned in valgus alignment in seven cases, in varus alignment in one case, and in a neutral (central) position in 22 cases (Figure 13). Cement filling was found to be adequate in all patients. Follow-up radiographs revealed no evidence of loosening, acetabular wear, or prosthetic dislocation.

## Discussion

Intertrochanteric fractures in elderly patients are frequently comminuted and markedly displaced. Due to underlying osteoporosis, these fractures are often associated with complications such as nonunion, implant failure, and femoral head perforation following internal fixation [10]. Although union rates approaching 100% have been reported in well-reduced, stable fractures treated with optimal implant positioning, failure rates of up to 56% have been observed in unstable or comminuted fractures, particularly in elderly patients with poor bone quality. The causes of fixation failure are multifactorial and include the initial fracture pattern, degree of comminution, suboptimal fixation, and compromised bone quality [11]. Fixation failure or inadequate fixation can result in complications such as atelectasis, pressure ulcers, pneumonia, and deep vein thrombosis, particularly in patients with multiple comorbidities [12]. Therefore, facilitating early ambulation remains a key consideration in determining the optimal management approach for unstable intertrochanteric fractures in osteoporotic elderly patients. Hemiarthroplasty serves as a widely accepted alternative treatment, providing early full weight-bearing. Moreover, the use of a prosthetic replacement helps avoid many of the complications commonly associated with internal fixation [13]. Bipolar hemiarthroplasty was designed to address the limitations of unipolar implants, notably acetabular wear and loosening. The dual-bearing construct distributes motion across two interfaces, reducing acetabular erosion and improving functional range of motion. Cemented fixation provides early stability, particularly beneficial in elderly patients with osteoporotic bone [14].

In 1974, Tronzo first reported the use of a long-stem Matchett-Brown endoprosthesis for primary hemiarthroplasty in unstable intertrochanteric femur fractures [15]. Subsequent studies have reported favourable outcomes with various prosthetic designs based on this concept.

Sancheti et al. reported favourable outcomes using primary hemiarthroplasty for unstable osteoporotic intertrochanteric fractures in elderly patients. In their series of 37 cases, early mobilisation, low complication rates, and good functional recovery were achieved, with a mean HHS of 84.8. They concluded that hemiarthroplasty provides stable fixation and allows for early rehabilitation in this high-risk population [16]. Xie and Zhou reported excellent outcomes with primary cemented bipolar hemiarthroplasty for unstable intertrochanteric fractures in elderly osteoporotic patients. In their prospective series of 277 cases, early mobilisation was achieved within three days, with minimal complications, including acetabular wear (1.1%) and aseptic loosening (1.4%). The mean HHS improved from 83.7 at six months to 90.3 at the final follow-up [17]. Rodop et al. treated 54 elderly patients with unstable intertrochanteric fractures using cemented bipolar hemiarthroplasty, and most patients were ambulatory within the first postoperative week, with no dislocations or aseptic loosening. At one year, 57% achieved excellent and 33% good outcomes based on the HHS. The authors concluded that bipolar hemiarthroplasty provides stable fixation and allows early rehabilitation with low complication rates in osteoporotic elderly patients [18]. In our study, fair to excellent outcomes were achieved in 75% of cases, which is consistent with the results reported in most previous studies. However, this proportion was lower than that reported by Xie and Zhou [17], who observed exceptional outcomes with bipolar hemiarthroplasty in the management of unstable intertrochanteric fractures. The variation in results may be attributed to differences in patient selection, surgical technique, rehabilitation protocols, or assessment criteria used across studies.

Several studies have highlighted the advantage of early mobilisation following bipolar hemiarthroplasty for unstable intertrochanteric fractures. Kumar et al. noted a mean mobilisation time of 5.4 days [19], while Xie and Zhou reported 3.3 days. Sinno et al. found that patients treated with bipolar hemiarthroplasty mobilised much earlier (1.26 days) compared to those managed with DHS (9.6 days) [20]. Similarly, Grimsrud et al. observed low rates of recumbency-related complications, supporting the benefits of early postoperative mobilisation [21]. In our study, the mean time to achieve full weight-bearing was 3.2 days. The early mobilisation protocol likely contributed to the absence of postoperative complications such as pressure sores, pneumonia, or deep vein thrombosis, as most patients were ambulatory soon after surgery. This finding underscores the importance of early rehabilitation in optimising functional recovery and minimising postoperative morbidity.

Geiger et al. reported that arthroplasty for trochanteric fractures was associated with higher intraoperative blood loss (1,050±700 ml) compared to DHS (409±360 ml) and proximal femur nail (PFN) (332±277 ml), reflecting greater surgical exposure and cement use. Although this increased transfusion requirement did not significantly affect postoperative mortality after adjusting for age and comorbidities, it should be balanced against the benefits of early mobilisation and lower revision rates in selected elderly patients [22]. Vandeputte et al. also reported higher intraoperative blood loss and transfusion needs with the endoprosthesis compared to the compression hip screw, due to greater surgical exposure and cement use. However, no significant differences were noted in operative time, complications, or mortality, suggesting the higher blood loss is acceptable in elderly osteoporotic patients who benefit from early mobilisation [23]. Xie and Zhou reported an average intraoperative blood loss of 225.8 ml for primary cemented hemiarthroplasty in elderly osteoporotic patients with unstable intertrochanteric fractures. These findings indicate that the procedure can be performed efficiently with relatively low blood loss, suggesting it is a safe and effective option for early mobilisation in this high-risk population. In this study, the average intraoperative blood loss was 361.03 ml, with only nine patients requiring blood transfusion. This reflects efficient surgical technique with relatively minimal blood loss and limited transfusion requirements, comparable to or favourable to previously reported studies on intertrochanteric fracture management.

Leg length discrepancy following hemiarthroplasty for proximal femur fractures is a known postoperative concern, though it is often minor and clinically insignificant in most cases [24]. Siwach et al. highlighted this issue in their study, reporting prosthesis sinking in two patients, with one experiencing more than 1.5 cm of shortening. They observed minimal discrepancy (<5 mm) in 64% of patients and limb lengthening between 5 and 10 mm in 28% [25]. Kumar et al. reported postoperative limb length discrepancy in a few patients following cemented bipolar hemiarthroplasty for unstable intertrochanteric fractures: four (20%) had shortening of less than 2 cm, two (10%) had shortening over 2 cm, and one (5%) had a lengthening of 1.5 cm. Despite these minor discrepancies, no functional limitation was noted [19]. In our study, limb length discrepancy was reported in 11 cases, with 10 patients showing shortening of less than 2 cm and one patient having shortening greater than 2 cm. In most cases (eight out of 11), the discrepancy resulted from excessive prosthetic subsidence due to inadequate calcar support, emphasising the importance of proper calcar reconstruction and stable fixation to prevent postoperative shortening. These findings highlight that meticulous intraoperative restoration of femoral length, along with precise implant positioning, calcar reconstruction, and stable fixation, is crucial to minimising postoperative limb length discrepancy and achieving optimal functional recovery.

Postoperative mortality following hemiarthroplasty for intertrochanteric fractures varies across studies depending on patient demographics and follow-up duration, but remains comparable to that reported for femoral neck fracture arthroplasties [2,3]. In a large cohort of 2,798 patients, the one-year mortality rate was 17.94%, rising to 29.76% at two years and 56.8% at five years. Mortality was notably higher in elderly patients, males, and those with multiple comorbidities. These outcomes suggest that the mortality associated with hemiarthroplasty reflects the underlying frailty and health status of the patient population rather than the surgical intervention itself [26]. Kumar et al. concluded that mortality was significantly higher in the bipolar hemiarthroplasty group compared to the proximal femoral nailing group, suggesting that PFN is associated with lower postoperative mortality in elderly patients with unstable intertrochanteric fractures. However, considering the advanced age and multiple comorbidities in this population, the increased mortality is likely influenced by these cumulative factors rather than the surgical procedure alone [27]. In our series, the mortality rate was 16.6%, with all deaths occurring after 30 days but within six months and none directly related to the surgical procedure. This rate is comparable to the one-year mortality reported in various studies on hemiarthroplasty for intertrochanteric fractures [26]. The immediate stability provided by bone cement allows for early postoperative ambulation, which significantly enhances functional recovery and accelerates rehabilitation. Early mobilisation not only reduces the risk of complications associated with prolonged immobility but also contributes to improved overall quality of life in elderly patients.

Reduction and stable fixation of both the greater and lesser trochanters are critical for achieving favourable functional outcomes following hemiarthroplasty for intertrochanteric fractures, though they often present technical challenges [28]. The greater trochanter serves as the attachment site for the gluteus medius and minimus, whose inadequate fixation can result in persistent pain, abductor insufficiency, and a Trendelenburg gait. Previous studies have shown that an unfixed greater trochanter fragment gap of more than 2 cm leads to marked abductor weakness and poor outcomes [29]. Similarly, fixation of the lesser trochanter is essential, as it provides attachment for the iliopsoas. Its failure can cause gait disturbances, loss of soft tissue tension, and flexion weakness [30]. 

Limitations

This study has certain limitations. The sample size was relatively small, and although all patients had a minimum follow-up of six months, with surviving patients followed up to 12 months and one-year outcomes analysed where available, longer follow-up would be required to comprehensively assess long-term outcomes. As this was a single-centre, non-comparative study evaluating a single treatment modality, direct comparison with internal fixation was not possible. Consequently, the limited sample size and follow-up duration restricted the assessment of long-term complications such as periprosthetic fracture, acetabular wear, and aseptic loosening.

## Conclusions

Primary cemented bipolar hemiarthroplasty is a safe and effective option for unstable intertrochanteric fractures in elderly osteoporotic patients, offering immediate stability, early weight-bearing, and faster functional recovery. Its limitations include greater surgical exposure, higher blood loss, and risks such as trochanteric nonunion and prosthetic subsidence, which can be reduced with meticulous technique and calcar reconstruction. In contrast, although internal fixation preserves the native joint and involves less surgical exposure, its effectiveness in this population is often limited by poor bone quality, higher failure rates, and delayed mobilisation.
